# Mitotic Catastrophe in BC3H1 Cells following Yessotoxin Exposure

**DOI:** 10.3389/fcell.2017.00030

**Published:** 2017-03-31

**Authors:** Mónica Suárez Korsnes, Reinert Korsnes

**Affiliations:** ^1^Department of Chemistry, Biotechnology and Food Science, Norwegian University of Life SciencesÅs, Norway; ^2^Nofima ASÅs, Norway; ^3^Norwegian Defence Research EstablishmentKjeller, Norway; ^4^Norwegian Institute of Bioeconomy ResearchÅs, Norway

**Keywords:** mitotic catastrophe, yessotoxin, single cell tracking, cancer, aneuploidy, checkpoint kinases, p-53, DNA damage

## Abstract

The marine toxin yessotoxin (YTX) can cause various cytotoxic effects depending on cell type and cell line. It is well known to trigger distinct mechanisms for programmed cell death which may overlap or cross-talk. The present contribution provides the first evidence that YTX can cause genotoxicity and induce mitotic catastrophe which can lead to different types of cell death. This work also demonstrates potential information gain from non-intrusive computer-based tracking of many individual cells during long time. Treatment of BC3H1 cells at their exponential growth phase causes atypical nuclear alterations and formation of giant cells with multiple nuclei. These are the most prominent morphological features of mitotic catastrophe. Giant cells undergo slow cell death in a necrosis-like manner. However, apoptotic-like cell death is also observed in these cells. Electron microscopy of treated BC3H1 cells reveal uncondensed chromatin and cells with double nuclei. Activation of p-p53, p-H2AX, p-Chk1, p-ATM, and p-ATR and down-regulation of p-Chk2 indicate DNA damage response and cell cycle deregulation. Micronuclei formation further support this evidence. Data from tracking single cells reveal that YTX treatment suppresses a second round of cell division in BC3H1 cells. These findings suggest that YTX can induce genomic alterations or imperfections in chromosomal segregation leading to permanent mitotic failure. This understanding extends the list of effects from YTX and which are of interest to control cancer and tumor progression.

## 1. Introduction

The marine toxin yessotoxin (YTX) is well known to induce various cytotoxic effects including apoptotic and non-apoptotic cell death. The present work broadens this knowledge providing the first evidence of genotoxic effects from it which may constitute a dominant and underlying cause of various observed types of cell death from YTX exposure. An intention for this work is also to lay out a case study to explore individual cell tracking as a tool in toxicological research. Individual cell tracking is a developing technology which can potentially provide fast diagnosis of changes in cell populations for example due to toxic insults. It can also help to guide hypothesis formulation in pilot studies.

This study shows results from exposing BC3H1 cells to YTX where they due to a spatially sparse distribution have minor contact inhibition of proliferation. Individual cell tracking indicates that YTX exposure then seems to lock the cells into an anti-proliferative state after their first division. This observation is the origin of the present hypothesis that YTX can be genotoxic and in this way has possible medical interest. Evaluation of the hypothesis includes check for additional traits of mitotic catastrophe which can be looked at as a strategy for higher eukaryotes to eliminate mitosis-incompetent cells (Vitale et al., 2011). These additional traits are from biochemical analyses as well as qualitative morphological inspections.

Genomic alterations throughout the cell cycle lead to genomic instability due to increased rate of spontaneous mutations promoting resistance to cell death as occurs early in tumorigenesis (Denisenko et al., 2016). Accumulation of such alterations can favor malignant cells to expand in stressful conditions (Hanahan and Weinberg, 2011). Genetically altered cells tend during division to develop abnormalities such as polyploidy, multipolar mitosis and aneuploidy (Nowell and Croce, 1986; Storchova and Pellman, 2004; Storchova and Kuffer, 2008; Donnelly and Storchová, 2015). These abnormalities are interconnected and can lead to mitotic catastrophe which can be considered as a regulatory mechanism to protect an organism (Denisenko et al., 2016).

Mitotic catastrophe has been defined as an oncosuppressive mechanism to avoid genomic instability. It can lead to a number of different outcomes, depending on the trigger and the cellular context (Weaver and Cleveland, 2005; Vakifahmetoglu et al., 2008; Vitale et al., 2011; Galluzzi et al., 2012). The mechanism can activate after detection of imperfections in the segregation of genetic material between daughter cells. This can result in multinucleated (mostly binucleated) tetraploid cells carrying a constant mitotic failure. Cells typically respond to such failures by inducing an irreversible cell death fate being apoptosis, necrosis or senescence (Vitale et al., 2011). Healthy cells progress trough all the four (G1, S, G2, and M) phases of the cell cycle, whereas cells bearing defects in the mitotic apparatus cannot proceed through the M phase and enter into activation of mitotic catastrophe. Mitotic catastrophe therefore includes some degree of mitotic arrest of duration critical for further cell fate (Vitale et al., 2011).

The mitotic checkpoint known as the spindle assembly checkpoint (SAC) ensures proper attachment of the chromosomes to the spindle microtubules allowing correct segregation of them. Prolonged SAC activation can lead to mitotic arrest but it may not always lead to mitotic catastrophe. Some cells can undergo mitotic catastrophe via mitotic slippage which is a phenomenon occurring when cells complete mitosis without SAC checkpoint. Multipolar mitosis can result from this phenomenon giving three or more daughter cells (Vitale et al., 2011).

Mitotic catastrophe initiates by perturbation in the correct segregation of the chromosomes between daughter cells, resulting in abnormal mitosis associated with spontaneous premature chromosome condensation, asymmetric distribution of chromosomes in the metaphase and the formation of giant cells with multiple micronuclei (Ianzini and Mackey, 1997; Castedo et al., 2004; Vakifahmetoglu et al., 2008; Kroemer et al., 2009). Formation of these cells was first believed to result from fusion between mitotic cells and cells in the S- or G2 phase and not as a consequence of daughter cell fusion. Endocycling has been proposed as the main mechanism forming giant cells, which can be either mono- or multinucleated depending on the starting point of the forming process (Erenpreisa et al., 2005). However, endocycling and formation of giant cells may represent an important cell repair and survival response as an additional mechanism of resistance after a genotoxic insult (Coward and Harding, 2014). The morphology of cells undergoing mitotic catastrophe resembles some features of apoptosis and necrosis but with some differences such as chromosomal breaks, clumps of chromatin and deficient karyokinesis (Castedo et al., 2004; Vitale et al., 2011).

The molecular pathways regulating mitotic catastrophe, share biochemical markers of apoptosis, necrosis and senescence (Roninson et al., 2001; Castedo et al., 2004; Vakifahmetoglu et al., 2008; Vitale et al., 2011). However, it has been difficult to characterize precisely the molecular pathways leading to mitotic catastrophe. This is due to the high degree of variability in the involved molecular cascades depending on expression and proper function of many proteins (Gascoigne and Taylor, 2009; Denisenko et al., 2016).

There is no consensus on the role of caspases during mitotic catastrophe. However, it is functionally linked to some of the biochemical markers of apoptosis. Activation of caspase-2 in response to DNA-damage, for instance, can occur both upstream and downstream of mitochondrial events such as mitochondrial membrane permeabilization, pro-apoptotic factors release, caspase-3 activation, DNA fragmentation and chromatin condensation (Lassus et al., 2002; Paroni et al., 2002; Read et al., 2002; Robertson et al., 2002). It has been reported activated in dying multinucleated cells (Vakifahmetoglu et al., 2008; Denisenko et al., 2016).

The tumor suppressor protein p53 plays a survival role by giving sufficient time to repair damaged DNA before the cells re-enter mitosis. It can probably mediate mitotic catastrophe by inhibiting proliferation of cells bypassing mitotic checkpoints. The protein suppresses chromosomal instability after mitotic arrest which may result in accumulation of polyploid cells (Dalton et al., 2010; Denisenko et al., 2016).

Activation of the checkpoint kinases Chk1 and Chk2 is considered a point of no return for cell cycle arrest (Topham and Taylor, 2013). Checkpoint kinases are activated when DNA damage or errors in cell cycle progression are detected. However, a conflict in cell cycle progression can lead to inactivation of the checkpoint kinases resulting in mitotic catastrophe (Bartek and Lukas, 2003a).

Several anti-tumor drugs, irradiation, micro-tubular poisons and actin blockers can induce mitotic catastrophe damaging chromosomes or perturb the mitotic apparatus through different phases of the cell cycle (Puck and Marcus, 1956; Tolmach and Marcus, 1960; Jordan and Wilson, 2004; Eggert et al., 2006; Gascoigne and Taylor, 2008; Vitale et al., 2011). A considerable fraction of tumor cells contain genomic alterations making them intrinsically more susceptible to mitotic aberrations. Inducers of mitotic catastrophe therefore appear to be interesting for cancer therapy.

Many authors have proposed therapeutic applications for the marine toxin YTX since it has a broad spectrum of cytotoxic effects (López et al., 2008, 2011; Korsnes, 2012; Alfonso et al., 2016; Tobío et al., 2016). However, its potential as a genotoxic compound has been poorly identified.

YTX is a small molecule marine polyether compound produced by the dinoflagellates *Protoceratium reticulatum* and *Gonyaulax grindleyi* (Murata et al., 1987; Satake et al., 1997, 1999; Draisci et al., 1999). Álvarez et al. (2016) recently reported *Gonyaulax taylorii*, as a new yessotoxins-producer dinoflagellate species found in Chilean waters. The toxin was initially isolated from the digestive gland of scallops *Pactinopecten yessoensis* which gives the name to it (Murata et al., 1987). It has been reported in different species of mussels including Spain, Norway, Italy, the Adriatic Sea, Russia, Chile and New Zealand (Paz et al., 2008).

YTX can induce different cell death modalities through activation of caspase-dependent and caspase-independent signaling pathways (Korsnes and Espenes, 2011; Korsnes et al., 2011; López et al., 2011; Tobío et al., 2012; Alonso et al., 2013). Cytotoxic effects vary significantly among cells exposed to it, depending on concentration and time of exposure (de la Rosa et al., 2001; Leira et al., 2002; Malaguti et al., 2002; Alfonso et al., 2003; Franchini et al., 2004; Callegari and Rossini, 2008; Ronzitti and Rossini, 2008; Orsi et al., 2010; Tubaro et al., 2010; Martín-López et al., 2012; Pang et al., 2014; Fernández-Araujo et al., 2015; Ferron et al., 2016). Anti-allergic and anti-tumoral activities inhibiting melanoma tumor growth and subacute immunotoxicity has been recently reported (Tobío et al., 2016; Ferreiro et al., 2017).

YTX appears to target some specific subcellular compartments such as the mitochondria, lysosomes and ribosomes (Bianchi et al., 2004; Korsnes et al., 2006, 2014; Malagoli et al., 2006). Activation of stress responses and cross-talk among cellular signaling pathways have been reported in cells under YTX treatment (Korsnes, 2012; Tobío et al., 2012; Korsnes et al., 2014, 2016; Rubiolo et al., 2014).

Korsnes and Korsnes (2015) demonstrate variability in lifetime distributions of single cells exposed to YTX. They showed that a small fraction of cells withstand the exposure much more than others, whereas some cells die long before the majority. The presence of such minorities might have interest for assessments of long term effects of YTX.

Young et al. (2009) are among the few reporters of genotoxic effects from YTX. They showed that YTX exposure in HepG2 cells during 3 h, affects some of the genes involved in the cell cycle, chromatin organization and DNA replication. Rubiolo et al. (2014) also showed that ER-stress induced by YTX treatment in glioma cells can arrest the G2/M phase and finally induced autophagic cell death.

The present work documents genotoxic effects leading to mitotic catastrophe in BC3H1 cells exposed to YTX. This attribute has well known medical interest. Cell death following mitotic catastrophe might be an effect of genetic instability generated by YTX exposure. Treatment with YTX induces formation of aneuploidy and/or polyploid cells. These cells are facing the mitotic-death programme ending in apoptosis-like or necrosis-like death or going to an apparent irreversible senescence. Mitotic catastrophe appears to be a highly desirable therapeutic endpoint to induce lethal instability in cancer cells. The potential to use YTX to induce mitotic catastrophe and target cell proliferation increases the list of its potential therapeutic applications.

## 2. Materials and methods

### 2.1. Toxin

YTX was obtained from the Cawthron institute (Nelson, New Zealand). YTX was dissolved in methanol as a 50 µm stock solution. The stock solution was diluted in Dulbecco's modified Eagle's medium (DMEM, Sigma), achieving a final concentration of 100 nm YTX in 0.2% methanol. Treated cells were incubated with 100 nm YTX and control cells were incubated with 0.2% methanol as vehicle. Control cells and treated cells were exposed to different end points (24, 48, and 72 h).

### 2.2. Cell culture

BC3H1 cell lines were isolated from primary cultures derived from mouse (ATCC Number CRL-1443). Recent data suggest that BC3H1 cells closely resemble cells in an arrested state of skeletal muscle differentiation than smooth muscle cells. BC3H1 cells are easy to grow as monolayer and they have shown responsiveness, long cell life span and stability during YTX treatment. They were purchased from the American Type Culture Collection (Manassas, USA). BC3H1 cells were cultured in Dulbecco's modified Eagle's medium (DMEM, Lonza, Norway) supplemented with 20% fetal calf serum (FCS, Bionordika, Norway). Cells were maintained undifferentiated at 37°C in a humidified 5% CO_2_ atmosphere.

### 2.3. Time-lapse video microscopy and single cell tracking

BC3H1 cells were plated onto 96 multiwell black microplates (Greiner Bio-One GmbH, Germany) for time-lapse imaging. Cells were cultured in medium (DMEM with phenol red, containing and 20% fetal bovine serum). Cells were imaged into Cytation 5 Cell Imaging Reader (Biotek, USA), with temperature and gas control previously set to 37°C and 5% CO_2_ atmosphere. Sequential imaging of each well was taken using 10x objective. The bright and the phase contrast imaging channel was used for image recording. A continuous kinetic procedure was chosen where imaging was carried out with each designated well within an interval of 5 min for a 96 h incubation period. All cells initially inside a 580 × 580 µm square were subject to tracking during 30 h using the experimental Kobio_Celltrack system^1^.

### 2.4. Transmission electron microscopy

BC3H1 cells were harvested and fixed with 2.0% paraformaldehyde and 1.25% glutaraldehyde in sodium cacodylate buffer (0.1 M, 7.2 pH) for 3 h at room temperature. After fixation the cells were washed with cacodylate buffer postfixed with 1% osmium tetroxide in cacodylate buffer for 1.5 h at room temperature. BC3H1 cells were washed with cacodylate buffer, and embedded in 3% low melting agarose. Subsequently, the cells were washed thoroughly in cacodylate buffer and dehydrated with a graded ethanol series (50, 70, 90, and 96% and four times 100%) 12 min for each step. The cells were then embedded in LR White resin (London Resin Company EMS, England). Ultrathin sections were obtained with an ultramicrotome (LEICA EM UC 6) and sections were picked up with formvar- and carbon-coated slot copper grids. Counterstaining was performed with 4% aqueous uranyl acetate and 1% KMNO_4_ for 10 min. The sections were examined with a FEI Morgani 268 transmission electron microscope at an accelerating voltage of 80 kW, and micrographs were recorded on a Veleta camera.

### 2.5. Phase contrast microscopy

3 × 10^5^ control and YTX treated cells were fixed in 4.0% paraformaldehyde 7.3 pH for 15 min. In brief, after fixation, cells were washed 3 times with PBS. Two drops of NucBlue Live ReadyProbes (Termofisher, USA) was added to a 1 ml Live cell imaging solution (Termofisher, USA). The prepared solution was added to the cells and incubated for 7 min at room temperature. Cells were then washed two times wih Live cell imaging solution (Termofisher, USA). Cells were observed within an Zeiss LSM 700 microscope and analyzed using the phase contrast optics and DAPI filter. All images were taken using a Plan Apochromat 20 × 0.8 ph2M27 objective.

### 2.6. Micronuclei visualization using fluorescence microscopy

1 × 10^4^ control and YTX treated cells were fixed in 4.0% paraformaldehyde 7.3 pH for 30 min at room temperature. After fixation, cells were washed 3 times with PBS. The fixative was then replaced with prewarmed Live cell imaging solution containing 50 nm LysoTracker red DND-99 (Life Technologies), and the cells were further incubated for 30 min at 37°C. Cells were analyzed with a Leica confocal laser scanner microscope SP5 (Leica Microsystems Wetzlar GmbH, Wetzlar, Germany).

### 2.7. Flowcytometry

2 × 10^6^ control and YTX treated cells were harvested and collected by centrifugation. In brief, cells were resuspended in 1 ml 1X PBS 7.2 pH and fixed in 4.0% paraformaldehyde for 10 min at 37°C. Cells were chilled on ice for 1 min. The fixative was removed prior to permeabilization by centrifugation and the cells were permeabilized by adding ice-cold 90 min methanol. Cells were incubated for 30 min on ice. 1 × 10^5^ cells were resuspended in 500 µL incubation buffer (0.5% BSA in 1X PBS) and rinsed two times by centrifugation. Cells were blocked in incubation buffer for 10 min at room temperature and incubated with p-p53 (Ser15), p-H2AX (ser139), p-Chk1 (Ser345), p-Chk2(Thr68), p-ATM (Ser1981), and p-ATR (Ser428) antibodies (Upstate, Cell Signaling USA), working dilutions 1:500 for 1 h at room temperature. Cell were then washed by centrifugation in 2 ml incubation buffer. Cells were incubated in fluorochrome-conjugated secondary antibody fluorescein (FITC)-conjugated donkey anti-rabbit (1:1,000, Jackson Immuno Research, USA) diluted in incubation buffer for 30 min at room temperature. Cells were washed by centrifugation in 2 ml incubation buffer and finally resuspended in 0.5 ml PBS and analyzed using a MACSQuant analyzer (Miltenyi Biotec GmBH, Bergisch Gladbach, Germany), following the manufacturer's instructions. The fluorescence of the FITC probe was analyzed using 495 nm excitation and 519 nm emission wavelengths. Statistical analysis were performed using LibreOffice Calc providing computation of confidence intervals.

## 3. Results

**Figure 1:**
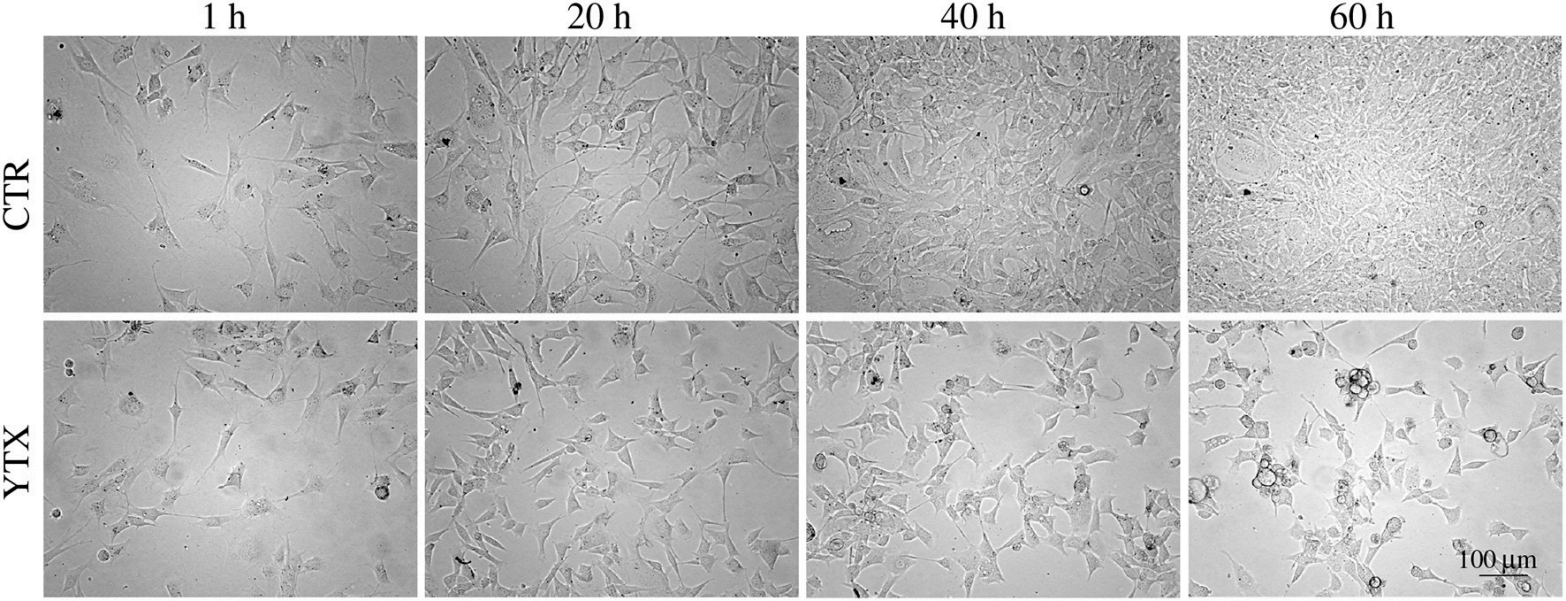
**Images of BC3H1 cells after exposed to 100 nM YTX for 1, 20, 40, and 60 h (lower row)** as compared to control cells **(upper row)**. These data simply indicate that YTX makes the cells stop to divide before 20 h. The images are representative for more than three independent experiments.

Simple visual inspection of continuous time-lapse video of living cells in a test well (Petri dish) shows that cell division mainly stops before 20 h after start of YTX exposure (Figure 1).

**Figure 2:**
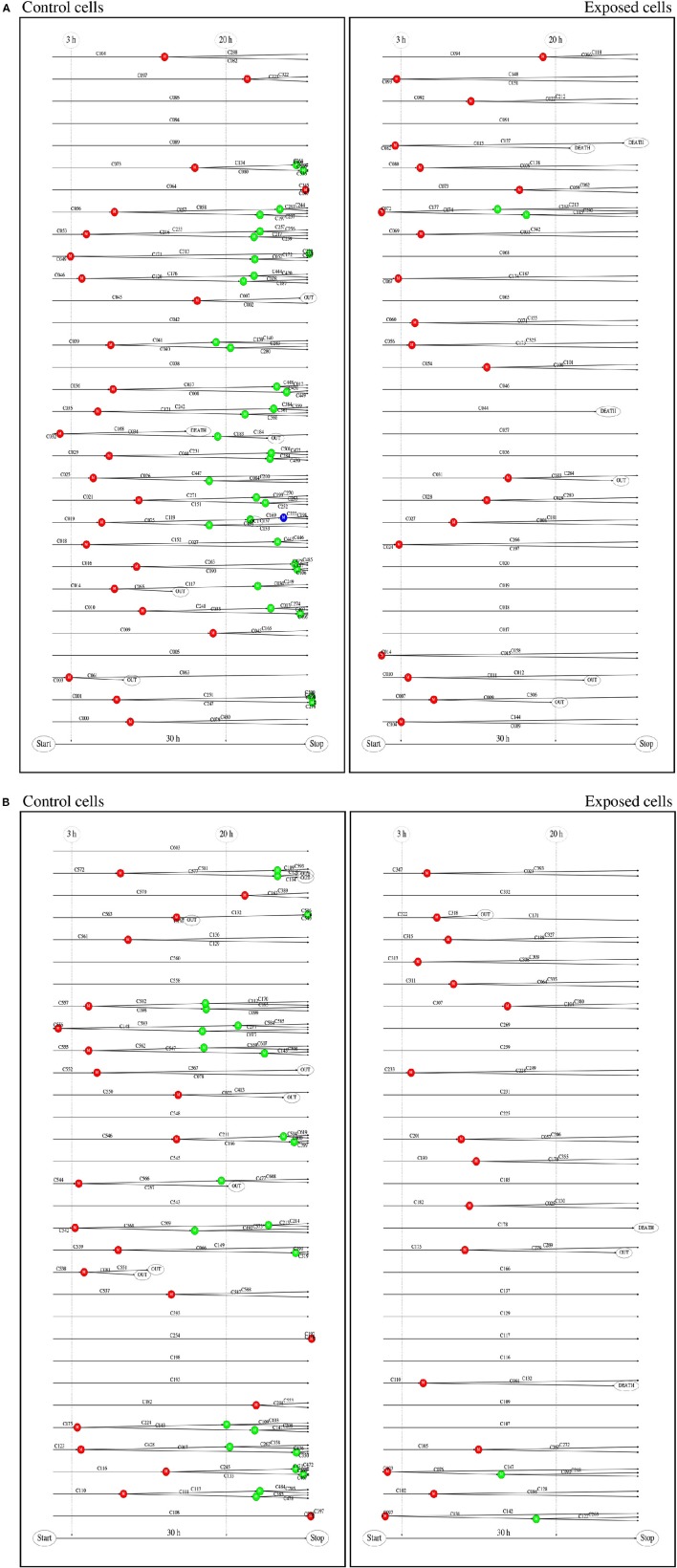
**(A)** Pedigree trees from single cell tracking. These data are for all cells initially inside a 580 × 580 μm square during 30 h at start of exposure. This gave 62 (51 complete) and 61 (56 complete) pedigree trees for the control and exposed cells respectively (cf. second part of this figure). Twelve of the initial (root) control cells did not divide during the observation period and 27 exposed cells similarly did not divide during that time. One control cell and two exposed cells were excluded from this statistics since they moved out of the recorded area (without division) before 15 h from the start. **(B)** Red, blue, and green bullets, respectively represent first, second, and third generation of cell division. Note that exposed cells tend only to divide one time (red bullet) if they do not divide shortly after start of exposure. Exposed cells din not divide after 20 h. Detailed visual inspection and biochemical measurements of DNA damage signaling checkpoints support the idea that permanent damage may take place in exposed cells during the first cell division.

**Figure 3:**
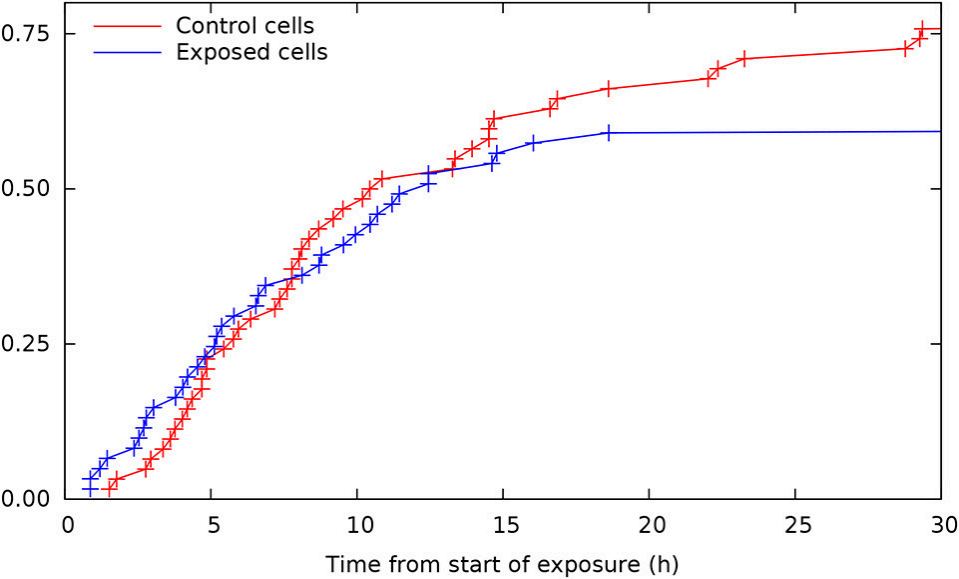
**Cumulative distributions for time from start of exposure to first cell division for control and exposed cells (derived from individual cell tracking)**. Note that exposed cells tend to divide at least as frequent as control cells up to about 8 h from start of exposure. This result illustrates potential methods for early detection of premature cell division based on more extensive cell tracking as demonstrated in the present work.

Single cell tracking brings details in this description. Figure 2 shows results from such tracking providing development of pedigree trees for the cells in a 580 × 580 µm square at start of exposure and the following 30 h (see enclosed Supplementary Data). There are 62 and 61 such pedigree trees for the control and exposed cells respectively. The figure shows that 15 of the control cells and 24 of the exposed cells did not divide (among which one died after almost 30 h). Note that exposed cells here tend to divide only one time after start of exposure (except when they divide very early). Four of the control cells did divide before 3 h from start of the experiment whereas 8 of the exposed cells did divide before that time. This simple observation may serve as a preliminary indication that YTX may accelerate the first cell division. Figure 3 further shows that exposed cells tend to divide at least as frequently as the control cells up to about 8 h from start of exposure.

**Figure 4:**
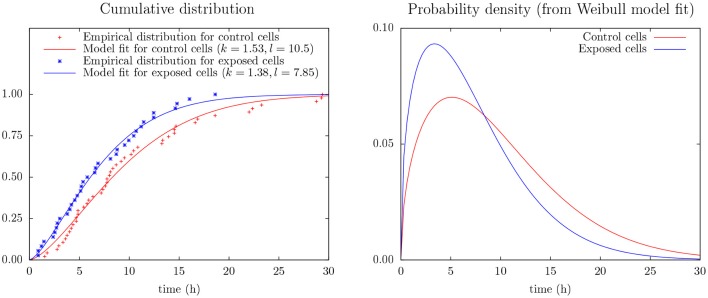
**(Left)** Empirical distribution and Weibull model fit for time duration from start of exposure to first mitosis of control and exposed cells as derives from cell tracking (cf. Equation 2). The data include only mitosis events during the first 30 h of observation. **(Right)** Probability density from the Weibull model fit (cf. Equation 1).

Figure 4 shows the (conditional) distribution of duration from start of exposure to the first cell division for the subset of cells actually dividing during the following 30 h (after start of exposure).

This figure includes results from an experiment to apply Weibull analysis of these data and which also indicates that YTX puts forward the first time cell division. Weibull analysis is a common approach to parameterize distributions of extremes or times of failures/lifetimes (Fisher and Tippett, 1928; Leadbetter et al., 2012). It simplicity (few parameters) potentially facilitates data greedy predictions beyond the capacity of pure empirical distributions. The model parameters are here for a two parameter Weibull probability density distribution:

(1) $$\begin{array}{l} f\left(t;\lambda ,k\right)=\left\{ \begin{array}{cc} \frac{k}{\lambda }{\left(\frac{t}{\lambda }\right)}^{k-1}e^{-{\left(\frac{t}{\lambda }\right)}^{k}}\; & \text{if }t\geq 0\\ 0\; & \text{otherwise} \end{array}\right. \end{array}$$

where *k* is a shape parameter and λ here defines time scale. The corresponding cumulative distribution is:

(2) $$\begin{array}{r} F\left(t;\lambda ,k\right)=1-e^{-{\left(\frac{x}{\lambda }\right)}^{k}} \end{array}$$

Inter-cellular influence can complicate statistical interference from experiments on cells in single test wells. The present few data (Figure 4) make it seem like YTX brings forward the first cell division after exposure or alternatively make some cells never divide (note *k* > 1.5 for the Weibull analysis of the control cells). However, note also that cells may need time to adapt to the experimental conditions at the start of an experiment.

Figure 2 indicates that some control cells tend to be slow to divide (or not divide at all). This possibility may have some biological meaning outside the scope of this work (for example facilitate unknown specialization or heterogeneity). However, possible existence of cells which are programmed not to divide (or are slow to divide), may respond differently to YTX exposure as compared to proliferating cells since cell division seems to be events of vulnerability.

Possible fat tail distributions of time for cell division among control cells is in practice difficult to observe. Hence, following the principle of Occam's razor (Domingos, 1998, 1999), the simplest models for it may guide interpretations of observations. The present observation that some cells do not divide during the first 30 h of monitoring, is consistent with the bold assertion of a positive fraction of non-reproductive cells in a maintained cell line. The following simple model illustrates that if for example 10% of the cells are found not to be reproductive (as a simple assumption from the present observations), then a cell division results in a non-reproductive cell with probability 5% in a stably maintained cell line population.

**Figure 5:**
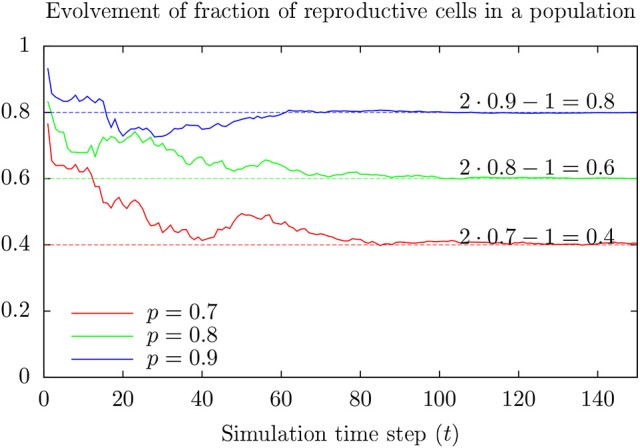
**Result from toy model showing development of the fraction *R*_*t*_ of reproductive cells at time *t* in a population where cells at birth are reproductive with probability *p* > 0.5**. Note that *R*_*t*_ approaches *R*_∞_ = 2*p* − 1 as time goes (faster for large values of *p*). This happens (almost always) for initially large enough numbers of reproductive cells.

Assume only a fraction of the cells in a population can divide, and for any time period of length △*T*, a reproducible cell in the population divide with a probability of for example 0.1. Figure 5 shows a possible time development of the fraction of reproductive cells in such a population for various values of *p*.

The fraction of reproductive cells tends to approach 2 *p* − 1 after some time (assuming *p* > 0.5). Appendix 1 in Supplementary Material provides a more intuitive model giving the same result which generalizes to reasons for the possibility that maintained cell line populations can include individuals of specially low proliferative capacity. These cells may respond differently to toxic exposure as compared to the majority since cell division represents a vulnerability.

**Figure 6:**
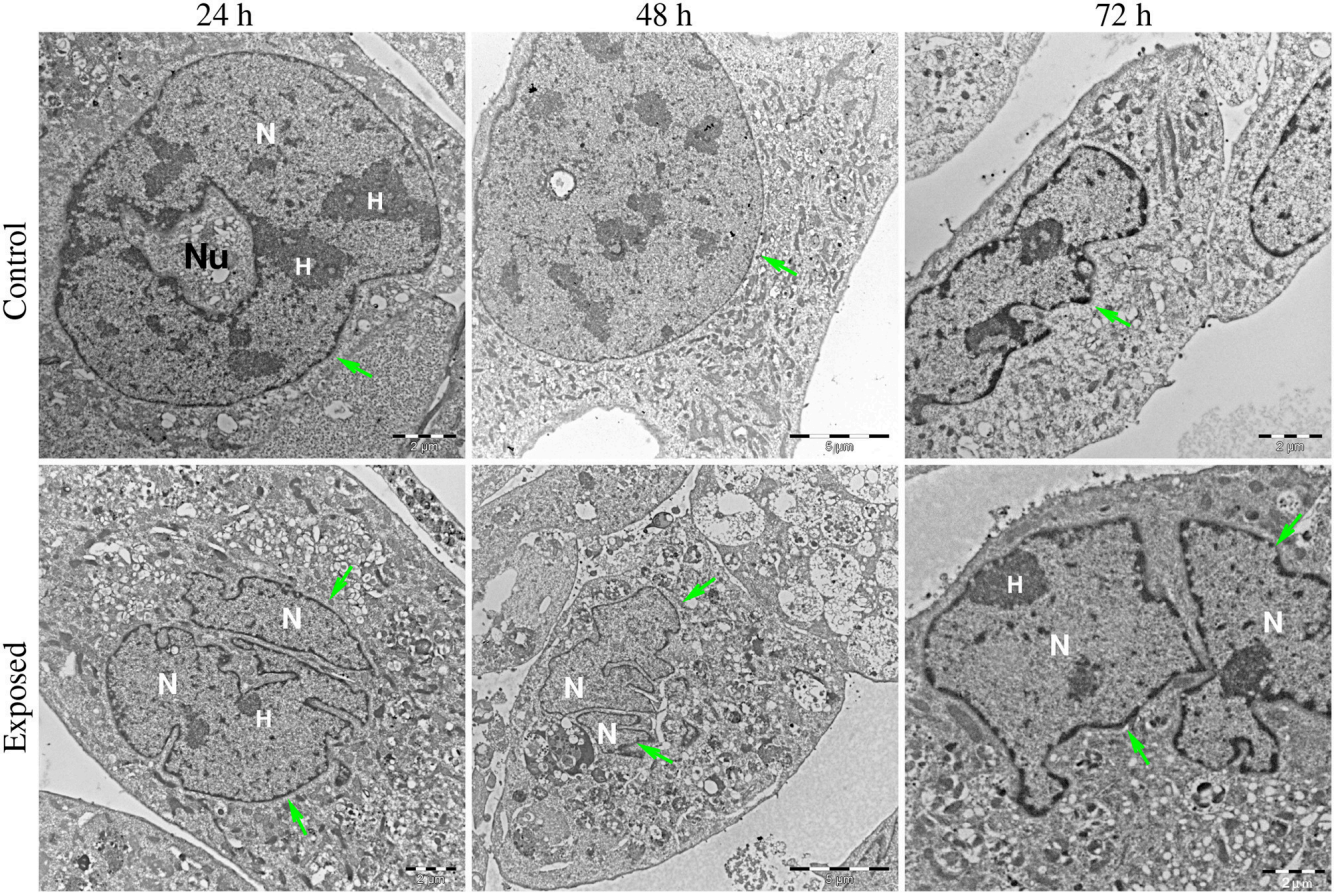
**Transmission electron microscopy images of BC3H1 cells exposed to 100 nM YTX for 24, 48, 72 h**. **(Upper row)** Shows control cells displaying normal nuclear morphology. **(Lower row)** Shows binucleation which is a morphological sign of mitotic catastrophe. N, Nucleus; H, heterochromatin; Nu, nucleolus. Green arrows points on nuclear envelop.

**Figure 7:**
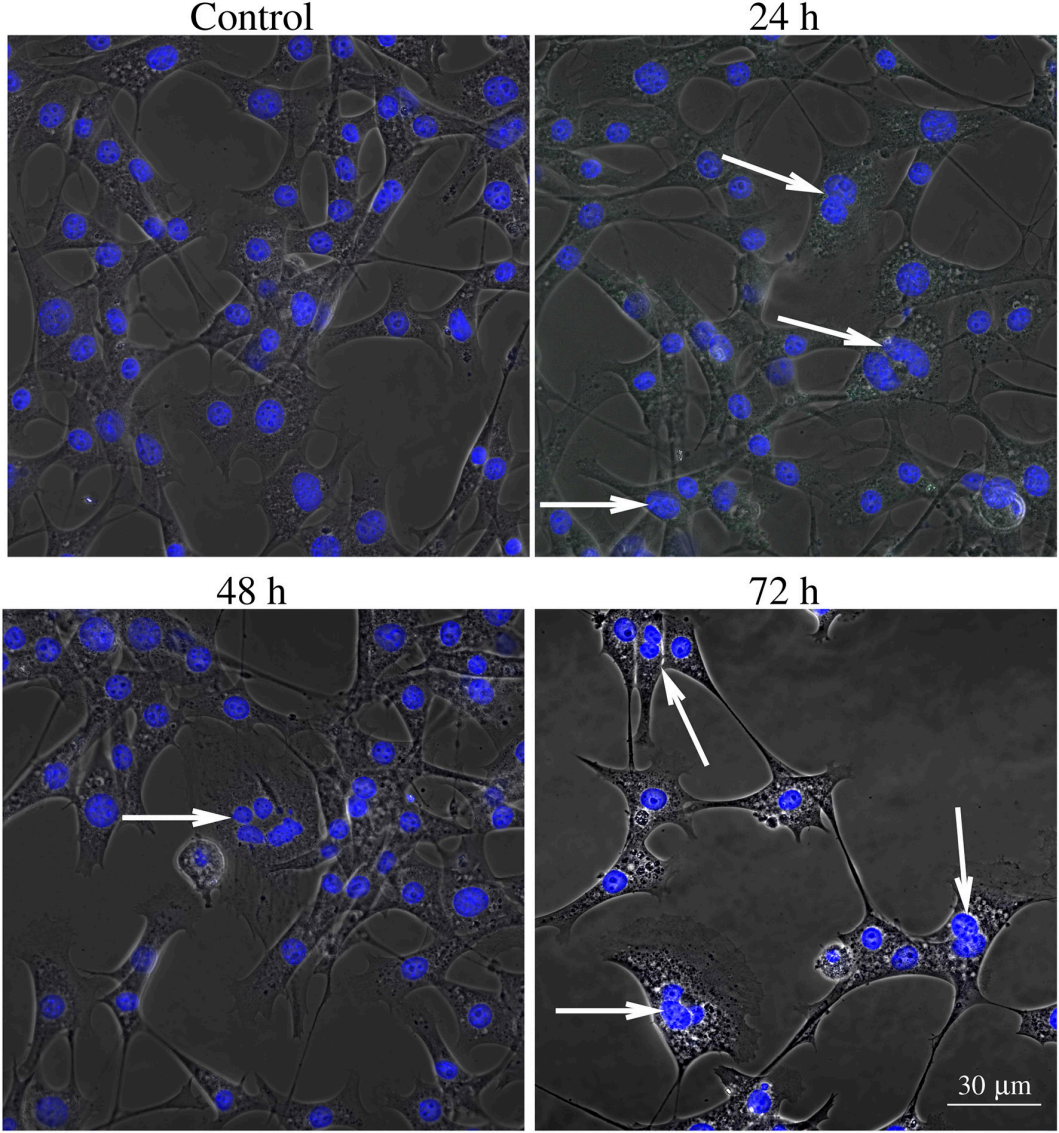
**Phase contrast image of BC3H1 cells exposed to 100 nM YTX for 24, 48, 72 h and stained with Hoechst (blue fluorescent color)**. White arrows show multiple nuclei in YTX-treated cells. The number of multinucleated and polyploid cells tends to increase upon YTX treatment.

**Figure 8:**
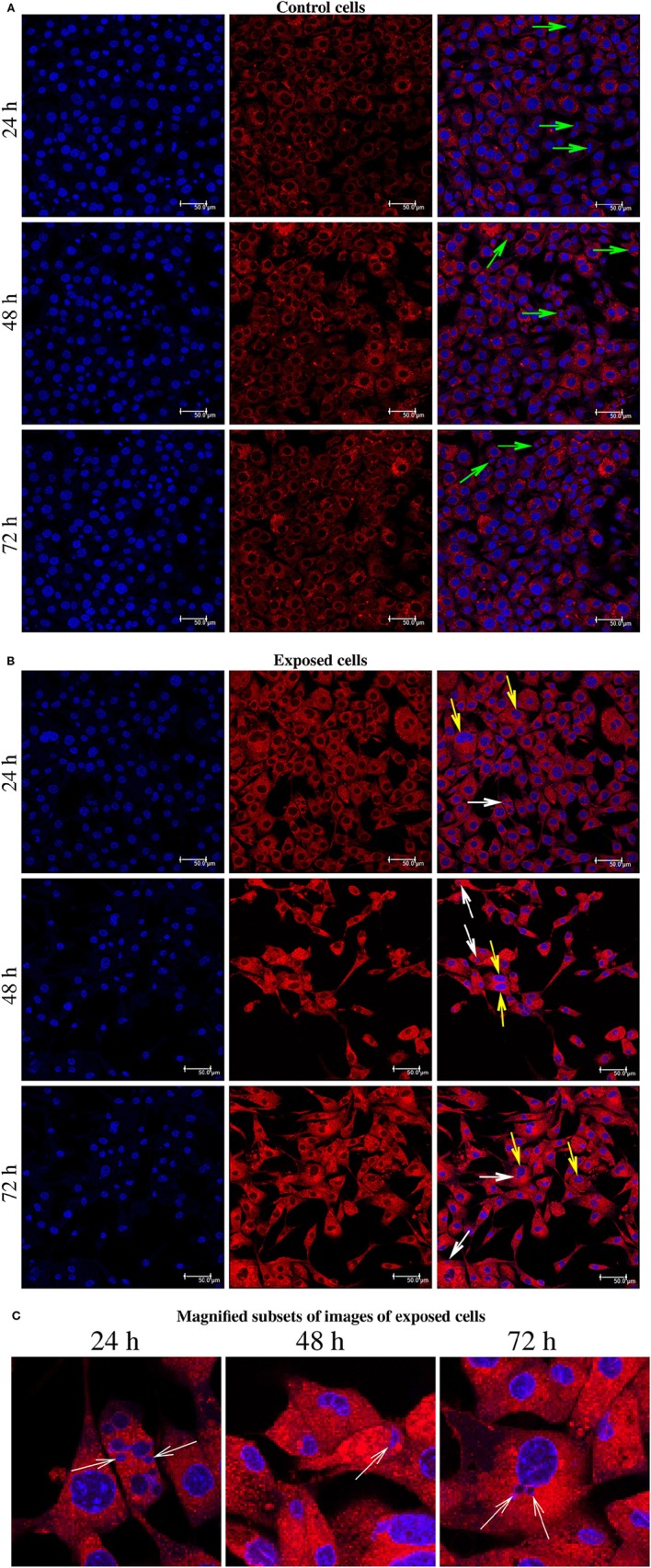
**(A)** Immunofluorescence labeling using Hoechst and LysoTracker Red DND-99 Staining for BC3H1 cells after exposed to 100 nM YTX for 24, 48, 72 h (first part is for control cells). Left columns: Hoechst labeling showing nuclei. Middle columns: Labeling with LysoTracker Red. Right columns: Two-color overlay with Hoechst and LysoTracker Red. Green arrows show the chromosomes lined up across the equator of the mitotic spindle. **(B)** White arrows point to the micronuclei. Yellow arrows show abnormal chromosomal segregation. **(C)** Magnified subsets from the color overlay images for exposed cells showing in detail the presence of micronuclei (white arrows) at 24, 48, 72 h of exposure. The results are representative for more than three independent experiments.

Figures 6–**8** show examples of the presence of nuclear alterations in cells after YTX exposure and which constitute the most prominent morphological characteristic of mitotic catastrophe (Vitale et al., 2011). Both the electron microscopy images (Figure 6) and the phase contrast images of colored cells (Figure 7) show multiple nuclei in treated cells.

Video recordings of control and exposed cells also show that YTX treatment tend to increase the occurrence of giant cells with multiple nuclei. This is a prominent morphological characteristics of mitotic catastrophe. Giant cells contain different sizes of nuclei with nuclear envelops around clusters of chromosomes or chromosome fragments (Nagl, 1990; Edgar and Orr-Weaver, 2001).

The present immunofluorescence labeling with the fluorescent dye Lysotracker red also help to reveal nuclear alterations in cells exposed to YTX (Figure 8). It reveals multi- and micronuclei. Micronuclei are cytoplasmatic bodies surrounded by nuclear membranes having incomplete chromosomes that were not successfully incorporated into a daughter cell during cell division. Their presence corroborates genetic damage and chromosomal instability (Fenech et al., 2011).

**Figure 9:**
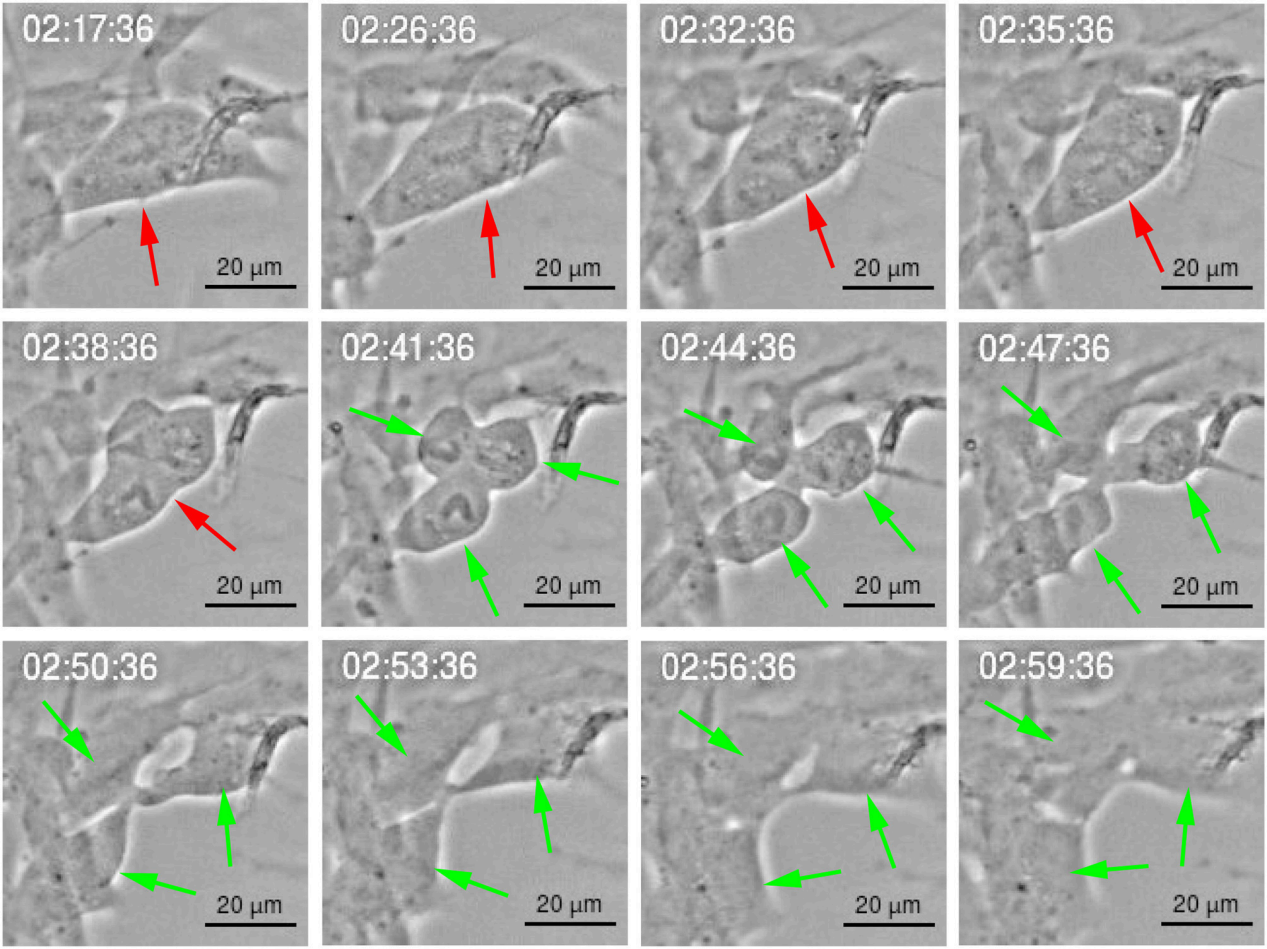
**Multipolar cell division of BC3H1 cell exposed to 100 nM YTX**. Asymmetric cell division showing generation of three daughter cells. Red arrow shows a cell before division. Green arrows show resulting daughter cells.

Figure 9 shows recordings of an example of asymmetric cell division under YTX treatment resulting in generation of three daughter cells which are most likely aneuploid.

Such cell division frequently lead to asymmetric distribution of the cytoplasm (anisocytosis), DNA (anisokaryosis) and chromosomes (aneuploidy). These events associated to mitotic catastrophe (Castedo et al., 2004).

**Figure 10:**
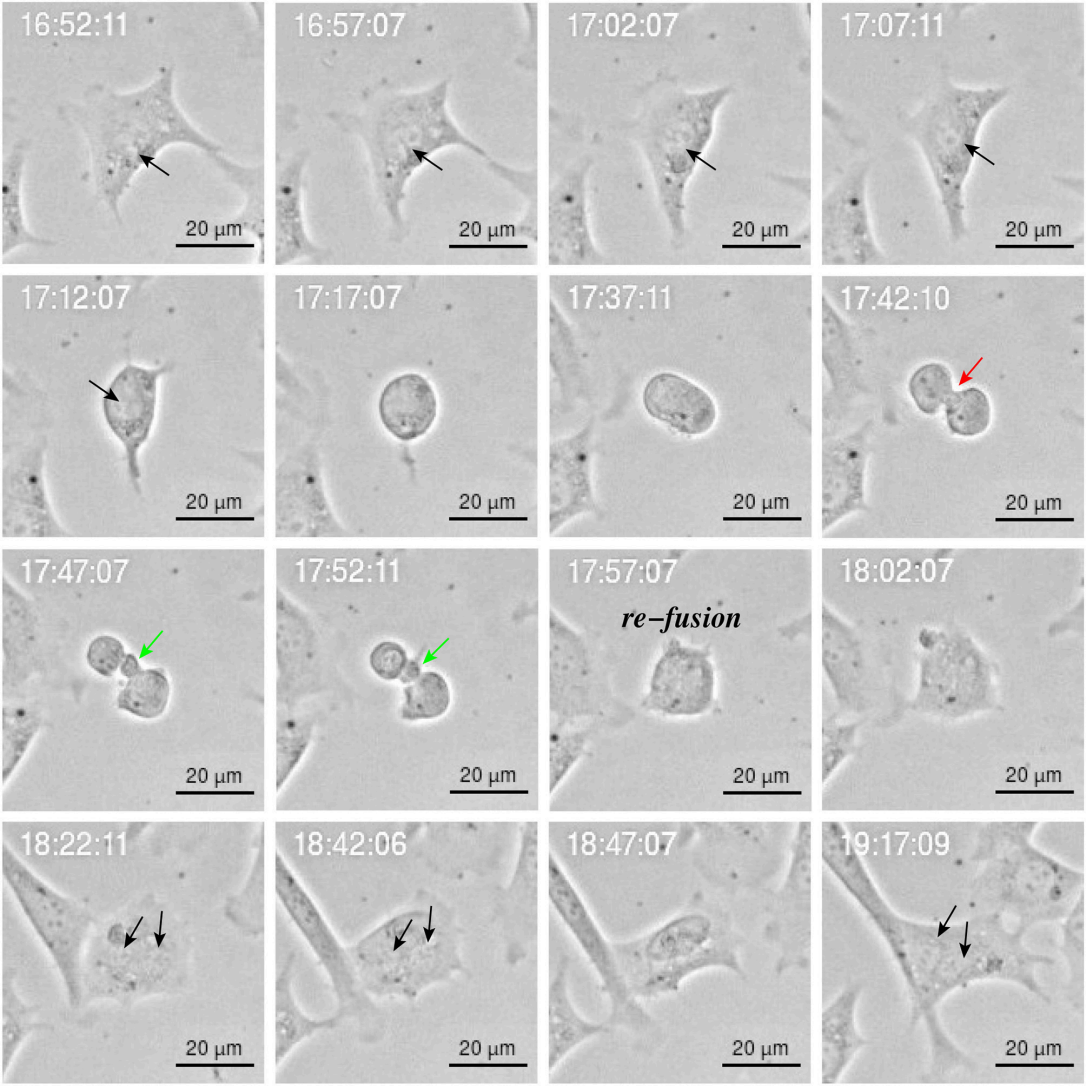
**Image sequence of YTX-treated BC3H1 cell showing asymmetric bipolar mitosis with incomplete cytokinesis including re-fusion of daughter cells**. Note the cleavage furrow (red arrow) and the midbridge (green arrow). Note that the final cell has double nucleus (**black arrow**).

Cytokinesis is the final step in cell division and is characterized by formation of a contractile ring which originates a cleavage furrow and a midbridge. It ends with the localization of the midbody in the middle of the midbridge which is cleaved to physically separate the daughter cells (Telentschak et al., 2015). Figure 10 shows failure in this process during YTX exposure (corroborating mitotic aberrations).

**Figure 11:**
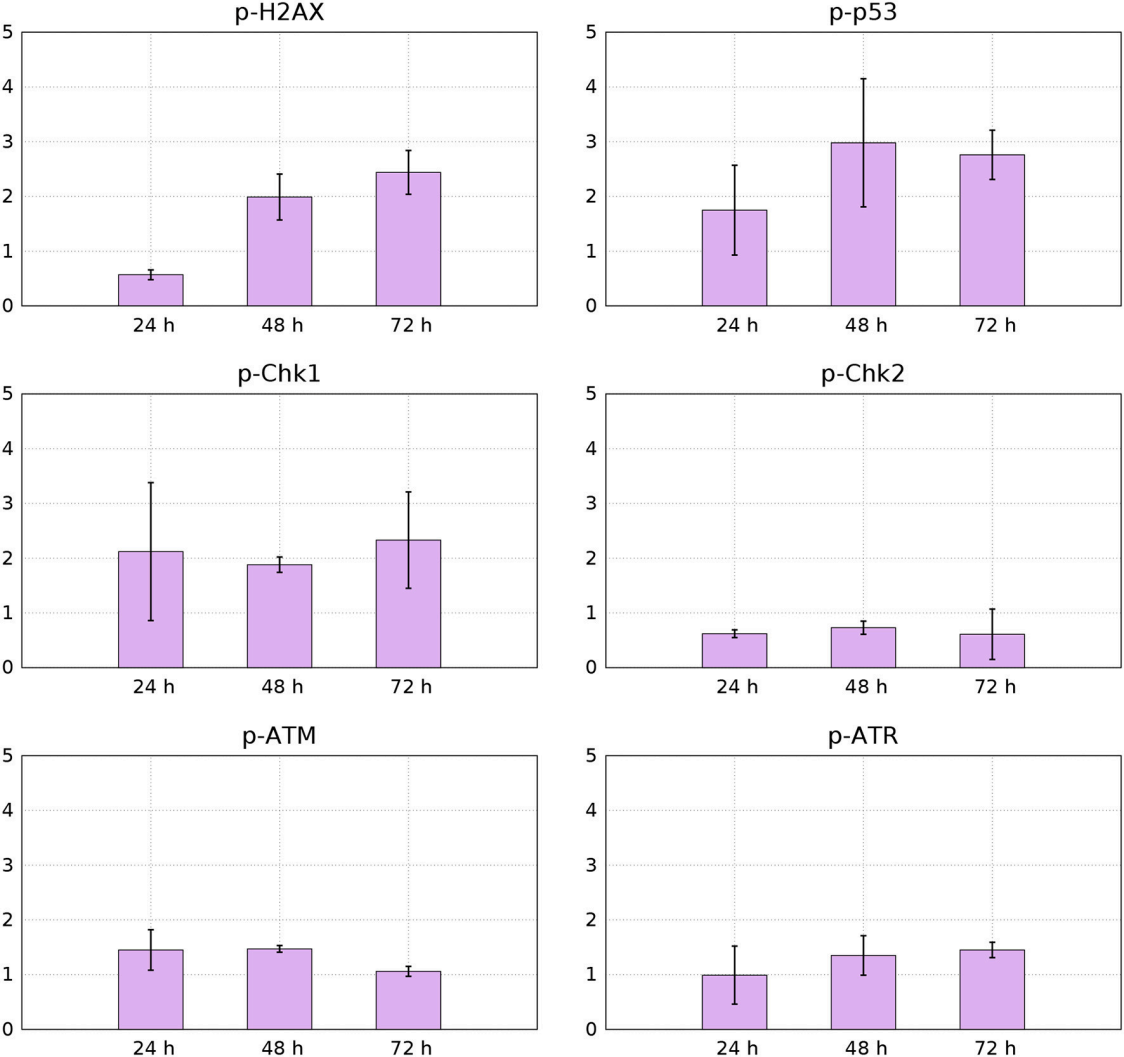
**Median values from 3 independent flow-cytometry measurements of FITC immunofluorescence signals for p-p53, p-H2AX, p-Chk1, p-Chk2, p-ATM, and p-ATR of YTX-treated cells relative to control cells (95% confidence intervals)**.

Figure 11 shows results from measurements of phosphorylation of the DNA structure checkpoint proteins H2AX, p53, Chk1, and Chk2 via flowcytometry based on indirect fluorescein isothiocyanate (FITC).

Phosphorylation of histone H2AX is necessary for proper condensation of chromatin Ichijima et al. (2005). It rapidly phosphorylates in response to DNA double strand breaks (Redon et al., 2002). Giant cells can therefore, as expected, contain increased levels of active H2AX (Storchova and Kuffer, 2008; Atsumi et al., 2011) Figure 11 shows down-regulation of active H2AX at 24 h treatment and increased levels of it at 48 and 72 h. This down-regulation of H2AX can indicate premature senescence, growth-arrest or maintenance of continuous quiescence under p53 regulation and stable-diploidy (Atsumi et al., 2011). BC3H1 cells might during the first hours of YTX exposure, be at a quiescent or premature senescence status having a stable diploidy causing the levels of H2AX to reduce.

Aberrant cell division in which cytokinesis failure is caused by disturbance of cleavage-furrow formation, results in accumulation of polyploid cells with increased amount of spontaneous DNA damage, abnormal mitosis and prolonged mitotic arrest. This can lead to accumulation of DNA double strand breaks (Storchova and Kuffer, 2008; Atsumi et al., 2011). H2AX phosphorylation may increase in cells which develop immortality subject to DNA replication stress (Atsumi et al., 2011).

YTX treatment increases phosphorylation of p53 in BC3H1 cells (Figure 11). This observation confirm DNA damage and induction of cell death. p53 can act as a tumor suppressor by inducing growth arrest and death of cells suffering DNA damage. Active p53 interacts with various cellular signaling pathways leading to distinct types of cell death. The mode of cell death modulated by p53 depends on the type of stress and duration as well as cell type (Denisenko et al., 2016).

Two protein kinase pathways are central to control cell cycle checkpoints to adjust the response to different types of DNA damage. They are the ataxia telangiectasia mutated (ATM) and ataxia telangiectasia and Rad3-related protein (ATR) sensor kinases. They phosphorylate the effector kinases Chk1 and Chk2 (Bartek and Lukas, 2003b). Chk1 is preferentially expressed in the S and G2 phases of the cell cycle and is phosphorylated by ATR (Vakifahmetoglu et al., 2008). Chk2 is expressed throughout the cell cycle and is phospohorylated and activated by ATM (McGee, 2015). However, ATM and ATR have different affinities for DNA structures in response to DNA damage. ATM senses damage in DNA ends, whereas ATR in SSBS. Figure 11 shows that YTX treatment increases ATR-Chk1 phosphorylation in a time-dependent manner. This indicates early response to DNA damage probably in response to SSBS.

Intracellular detection of p-Chk2 by flow-cytometry revealed down-regulation of this protein (Figure 11). This result might support premature mitotic entry and subsequent mitotic catastrophe as previously reported in HeLa syncytial model (Castedo et al., 2004). Chk2 activates during defects in cell cycle progression or DNA damage and arrests the cell cycle at the G2/M boundary (Nigg, 2001; Lukas et al., 2003). Castedo et al. (2004) demonstrated that mitotic catastrophe induced by DNA damage, relies on Chk2 inhibition and thereby increasing the number of apoptotic cells. Castedo et al. (2004) reported that multinucleated cells generated by fusion of non-synchronized cells, can generate multipolar divisions giving more than two daughter cells when Chk2 and caspases are simultaneously inhibited. Such fusion is therefore a candidate to explain observed down-regulation of Chk2 (Figure 11).

## 4. Discussion

This contribution brings the first evidence that YTX can induce genotoxic effects in BC3H1 cells leading to mitotic catastrophe at least if the exposure starts early enough in an exponential growth phase. Simple image recordings of cells during few days, show that YTX halts cell proliferation (Figure 1). This work also demonstrates the potential to gain information from tracking single cells from image recordings and which seems to be a valuable tool to observe cell fate (Korsnes and Korsnes, 2015; Skylaki et al., 2016) including detection of mitotic catastrophe (Rello-Varona et al., 2010).

Figure 2 shows that YTX suppresses a second round of cell division after exposure supporting the idea that significant DNA damage takes place during the first cell division. The tracking data also indicate that YTX puts forward the first cell division (Figures 3, 5). Premature mitotic failure can trigger damage resulting in an irreversible cell cycle arrest that also precludes the amplification of genomically unstable cells or cells in state of senescence as reported by Vitale et al. (2011). Visual inspection (Figures 6–8) qualitatively demonstrates DNA damage from YTX exposure. Biochemical measurements of DNA damage signaling checkpoints (Figure 11) also support the hypothesis that YTX generates DNA damage. Further quantitative analyses are required to assess the biological significance of these findings. Future applications of cell tracking and analyses of pedigree trees may help to quantify DNA damage and if it for example is directly related to cell division.

Cell tracking reveals that some control and exposed cells do not divide during the first 30 h after start of exposure (Figure 2). These cells might be less vulnerable to genotoxic compounds since they avoid effects on the integrity of the genetic material during cell division. They may therefore, for future development of cell tracking applications, represent an opportunity to obtain complementary information on cell response.

The integrity of DNA is continually challenged and maintained in living organisms. Eukaryotic cells have evolved the DDR (DNA Damage response) which is a highly regulated signaling network responding to DNA lesions. Unrepaired DNA lesions activates checkpoints proteins that are tightly coordinated with cell cycle progression and ensure proper timing of cellular events (Vitale et al., 2011). Sub-optimal repair of DNA can lead to mitotic catastrophe where an early step is premature or inappropriate entry into mitosis caused by genotoxic insult, physical stress or defective cell cycle checkpoints (Roninson et al., 2001; Vakifahmetoglu et al., 2008).

Cells bearing defects may proliferate once the repair is complete (Portugal et al., 2010). However, inhibition of cell cycle checkpoints may limit the time available for repair, thereby forcing the cells to prematurely progress through the S and G2 phase producing an aberrant mitosis (Swanson et al., 1995).

Mitotic checkpoints such as spindle assembly checkpoint (SAC) help to maintain the fidelity of mitosis ensuring accurate chromosome segregation. SAC senses that the kinetochores of sister chromatids successfully attach to spindle microtubules and are under adequate tension (Lampson et al., 2004). This condition occurs during normal phases of mitosis. However, when the chromosomes are not perfectly oriented or aligned SAC activation persists and halts the progression from the metaphase to the anaphase to prevent chromosome missegregation in cells with spindle defects. Prolonged SAC activation often leads to mitotic catastrophe (Vitale et al., 2011).

Cell tracking allows early recognition of aberrant cell divisions in BC3H1 cells exposed to YTX, which appears to disturb segregation of the chromosomes (second and third part of Figure 2). This effect may be similar to the effect of many microtubular poisons or actin blockers.

Microtubular poisons affect spindle formation and prevents chromosome segregation leading to mitotic catastrophe. They arrest the final step of mitosis inducing failure in cytokinesis by targeting the contractile ring which is important for physical separation of daughter cells (Eggert et al., 2006). The best known mitotic catastrophe inducers are the microtubule targeting agents (MTAs) also known as spindle poisons (Jordan and Wilson, 2004; Kavallaris, 2010; Salmela and Kallio, 2013).

YTX-treated cells often undergo asymmetric cell division (Figure 9) or daughter cells may fail to physically separate and subsequently undergo refusion probably due to cytokinesis failure (Figure 10). YTX seems to target the contractile ring, since the inter-cellular bridge do not manage to separate properly (Figure 10). It may stop contraction of the contractile ring formation avoiding that furrow ingression proceeds and creates an inter-cellular bridge which must be resolved to create two daughter cells. Improper location of the furrow cleavage may affect the equally distribution of the chromosomes and organelles to each daughter cell and cytokinesis failure may also contribute to the formation of polyploid giant cells (Eggert et al., 2006; Telentschak et al., 2015). These results support the hypothesis that YTX produce DNA damage.

YTX treatment induces asymmetry and multipolar cell divisions (Figure 9) supporting the idea that chromosomal segregation may go wrong as evidenced by the generation of three daughter cells. YTX might disrupt proper attachment to the spindle microtubules to the kinetochore of each sister chromatid or induce lack of tension. Misaligned chromosomes or lack of tension is sufficient to activate the spindle assembly checkpoint (SAC). Prolonged SAC activation prevents metaphase-anaphase transition which ensures the faithful inheritance of the genetic material to offspring. Prolonged SAC activation can also lead to mitotic catastrophe (Vitale et al., 2011).

YTX exposure tends to increase the number of giant cells with multiple nuclei and micronuclei (Figures 6–8). The presence of multinucleated giant cells with abnormal nuclei has been regarded as a lethal phenotype resulting from premature mitosis or failure to complete mitosis (Weinert and Hartwell, 1988; Molz et al., 1989; Chan et al., 1999; Nitta et al., 2004; Wang et al., 2004). However, formation of giant cells may also be considered as a mechanism to escape from severe genotoxic damage. It can generate a small subset of cells undergoing reductive divisions (Coward and Harding, 2014). These cell divisions, which are “meiosis-like” in nature, can enable a viable progeny with near-diploid chromosomes that are proliferative and competent to re-initiate tumor growth (Ianzini et al., 2009; Vitale et al., 2011).

Vitale et al. (2011) reported that in some special circumstances, the damage signal during abnormal mitosis can lead to mitotic catastrophe resulting in senescence, or irreversible cell cycle arrest precluding generation of genomic unstable cells.

YTX is known to be a cell death inducer in many cellular systems including a broad spectrum of cancer cells (Korsnes and Espenes, 2011; Korsnes, 2012; Alfonso et al., 2016). Cancer cells are in some cases particular sensitive to induction of mitotic catastrophe because they are more prone to mitotic aberrations, cell cycle deregulation and they fail to activate DNA damage checkpoints (Dixon and Norbury, 2002). Understanding the molecular mechanism that lead to mitotic catastrophe has therefore important implications for tumor prevention and treatment (McGee, 2015).

Induction of multidrug resistance in tumors as a consequence of treatment with chemotherapeutic drugs is one of the major problems in the failure of many forms of chemotherapy (Ruth and Roninson, 2000). Tumors usually consist of mixed populations of malignant cells, some of them are drug-sensitive while others are drug-resistant. Tumor response to chemotherapy, however, rises a population of cells that is committed to die through mitotic catastrophe or senescence specially when apoptosis is blocked. Mitotic catastrophe constitutes an important mechanism of tumor selectivity because it can elicit a more intense, longer-lasting effect *in vivo* (Roninson et al., 2001; Portugal et al., 2010). A wide variety of anticancer compounds are under screening and novel molecular compounds may become a potential therapeutic tools. YTX may in this context represent a promising anticancer compound that can bind to DNA, target the cell cycle and induces mitotic catastrophe. These approaches are effective to target processes in cancer treatment.

## Author contributions

MK conceived the study and conducted the laboratory experiments, RK made the computer programming; both authors analyzed the results and wrote the manuscript.

### Conflict of interest statement

The authors declare that the research was conducted in the absence of any commercial or financial relationships that could be construed as a potential conflict of interest.

## Footnotes

^1^https://www.korsnesbiocomputing.no/.
