# Supplementary material for: Mitotic Catastrophe in BC3H1 Cells following Yessotoxin Exposure
Source: Front Cell Dev Biol. 2017 Mar 31;5:30. doi: 10.3389/fcell.2017.00030 (PMC5374163; doi:10.3389/fcell.2017.00030)
Supplement: Supplementary file 1 [file Presentation1.pdf]

Supplementary Material: Mitotic catastrophe in BC3H1 cells following yessotoxin exposure

Mónica Suárez Korsnes * and Reinert Korsnes
*Correspondence:
Mónica Suárez Korsnes
monica.suarez.korsnes@nmbu.no

VIDEO ILLUSTRATING CELL TRACKING

The enclosed video files

`https://korsnesbiocomputing.no/tracking_control_cells1.mp4`

and

`https://korsnesbiocomputing.no/tracking_exposed_cells1.mp4`

cover $30\,\mathrm{h}$ continuous recording of respectively control and exposed cells in test wells. See paper. The square dots of various colours here tag cells which at start are in the red square of size $580\,\mu\mathrm{m} \times 580\,\mu\mathrm{m}$. The statistical results in the paper are derived from track data for these cells and their descendants. There are also circular (partly white) dots tagging cells in the video. These are auxiliary (typically incomplete) tracks for cells which are not in the red square at start (or among their descendants). The purpose of these tracks are to serve multi-target tracking.
