# Supplementary material for: Mitotic Catastrophe in BC3H1 Cells following Yessotoxin Exposure
Source: Front Cell Dev Biol. 2017 Mar 31;5:30. doi: 10.3389/fcell.2017.00030 (PMC5374163; doi:10.3389/fcell.2017.00030)
Supplement: Supplementary file 2 [file Presentation2.pdf]

Appendix

1 FRACTION OF REPRODUCTIVE CELLS IN A MAINTAINED CELL LINE POPULATION

The following simplistic model shows that if a cell in a maintained cell line population has probability $p \geq 0.5$ to be reproductive, there will be a fraction close to $R_\infty = 2p - 1$ of reproductive cells after some time (cf Section 3).

Assume M_t and N_t respectively represent the number of reproductive and not reproductive cells in a maintained population at time t. The reproductive cells divide at each time step $t = 0, 1, 2, \ldots$ forming two cells each with a probability of p to be reproductive. Hence (approximately) $M_{t+1} = 2M_t \cdot p$ and $N_{t+1} = 2M_t \cdot (1 - p) + N_t$. This gives for $\mathbf{S}_t = [M_t, N_t]^\top$ the recursion:

$$\mathbf{S}_{t+1} = \mathbf{A}\mathbf{S}_t$$

where

$$\mathbf{A} = \begin{bmatrix} 2p & 0 \\ 2(1-p) & 1 \end{bmatrix}$$

The fraction $R_t = M_t/(M_t + N_t)$ of reproductive cells will in this case also attract to the constant $R_\infty = 2p - 1$ (provided $M_0 > 0$ and $p > 0.5$). This is easily seen by considering the increase $\triangle\mathbf{S}_t = \mathbf{S}_{t+1} - \mathbf{S}_t$ at each step of iteration of Equation 3 :

$$\triangle\mathbf{S}_t = \mathbf{B}\mathbf{S}_t$$

where $\mathbf{B} = \mathbf{A} - \mathbf{I}$ has a zero second column ($\mathbf{I}$ is the identity matrix):

$$\mathbf{B} = \begin{bmatrix} 2p - 1 & 0 \\ 2(1-p) & 0 \end{bmatrix}$$

Hence the increase $\triangle\mathbf{S}_t$ has the same (constant) direction as the first column of $\mathbf{B}$ (considered as a vector). This gives that the ratio $R_t = M_t/(M_t + N_t)$ of reproductive cells gradually approaches $(2p - 1)/[2(1 - p) + (2p - 1)] = 2p - 1$.
